# Artificial intelligence in acne vulgaris patients

**DOI:** 10.1590/1806-9282.20260753

**Published:** 2026-10-05

**Authors:** Tuğba Atak, Tuba Betül Karadeniz

**Affiliations:** 1 Kırıkkale University, Faculty of Medicine, Dermatology Clinic – Kırıkkale, Türkiye.

**Keywords:** Artificial intelligence, Acne, Patient, Perspective

## Abstract

**OBJECTIVE:**

The aim of this study was to evaluate the experiences and confidence levels of acne patients using artificial intelligence tools. This study investigates whether patients view artificial intelligence as a standalone resource or a facilitator of physician collaboration to support the ethical implementation of healthcare technologies.

**METHODS:**

This cross-sectional, descriptive study included acne patients aged ≥18 years with internet access. Alongside physician-led clinical evaluations, a structured questionnaire was administered.

**RESULTS:**

Among 123 patients (81.3% female), 45.5% utilized artificial intelligence tools for general health purposes before consulting a physician, with acne-specific artificial intelligence usage being significantly driven by the perception of rapid access (r=0.490, p<0.001). Despite this interest, 67.5% insisted on professional validation for artificial intelligence advice, and notably, no significant correlation was found between clinical acne score and artificial intelligence utilization for acne management. Additionally, 55.3% of participants expressed concern about the risk of artificial intelligence making incorrect diagnoses, and 48.8% stated that they were reluctant to share personal data, such as photos, with these systems. Of those who prefer artificial intelligence, 53.7% use it for “rapid and easy access,” while 52% stated that they “would never use it without consulting a doctor.”

**CONCLUSION:**

The integration of artificial intelligence in dermatology necessitates a physician-led, transparent approach that prioritizes digital health literacy and ensures these tools serve as supervised supplements rather than a replacement for human-centered care.

## INTRODUCTION

Characterized by visible primary lesions and potential permanent scarring, acne is a prevalent inflammatory disease that imposes a substantial physical and psychosocial burden, profoundly impairing quality of life across both adolescent and adult populations^1^.

Artificial intelligence (AI), particularly deep learning, has significantly advanced healthcare across various specialties, including dermatology, by augmenting clinical expertise rather than replacing physicians. However, the ultimate clinical success of these technologies depends not only on technical accuracy but also on patient trust and perceptions^2,3,4,5^.

The majority of existing literature focuses on the algorithmic performance of AI, leaving a gap in our understanding of the attitudes and behaviors of the patients who use this technology. This study aims to directly investigate the experiences and confidence levels of acne patients using AI tools. Our findings will inform future patient education strategies by clarifying whether patients perceive AI as a simple information source or as a facilitator of physician collaboration. By documenting the patient perspective, this research provides the empirical evidence necessary to support the ethical and safe implementation of healthcare technologies.

## METHODS

This cross-sectional, descriptive study was conducted among volunteer patients diagnosed with acne at the Dermatology outpatient clinic of Kırıkkale University Faculty of Medicine between December 2025 and February 2026. Following a detailed explanation of the study objectives, informed consent was obtained from all participants. Data were collected via a purpose-designed, nongraded descriptive questionnaire, which underwent a pilot study to ensure clarity and comprehensibility. The survey instrument was developed by the investigators specifically for this study population and has not been utilized in previous publications.

Inclusion criteria required participants to be aged 18 years or older, possess adequate cognitive health to complete the survey, and have access to both the internet and a smart device (smartphone, tablet, or computer).

The patient questionnaire comprised six main sections: socio-demographic characteristics, clinical history, AI experience (frequency and primary objectives), the perceived impact of AI on the patient–physician relationship and consultation frequency, attitudes and trust regarding AI advice (adherence, necessity of dermatologist validation, and error concerns), and perceptions of data security and AI’s future role in acne management. The complete list of the survey questions is provided in Supplementary Appendix 1.

During the clinical assessment, the physician recorded the patients’ acne severity using the Global Acne Grading System (GAGS)^6^, acne type, the presence of scarring, and history of previous treatments.

The study was conducted in accordance with the ethical principles outlined in the Declaration of Helsinki. Participants’ identity information was kept completely anonymous, and their consent was obtained by written/verbal declaration that the data would be used solely for scientific purposes. The participants were reminded that they had the right to refuse to participate in the study or to withdraw at any stage. The study protocol was reviewed and approved by the Kırıkkale University Non-Interventional Clinical Research Ethics Committee (Decision No: 2025.12.13, Date: 10.12.2025).

### Statistical analysis

Statistical analysis was performed using IBM SPSS Statistics software (Version 20.0). The normality of continuous variables was assessed using the Kolmogorov-Smirnov test. Continuous data were expressed as mean±standard deviation (SD), while categorical variables were presented as percentages. Categorical variables were compared using the chi-square test. For continuous variables, Student’s t-test was utilized for parametric data, whereas the Mann-Whitney U and Kruskal-Wallis tests were employed for non-parametric comparisons. Pearson correlation analysis was conducted to examine the relationships between acne-related factors (e.g., AI error concern, age, and acne score). Statistical significance was set at p<0.05.

## RESULTS

Of the 123 patients participating in the study, 81.3% (n=100) were female and 18.7% (n=23) were male. The mean age was 23.37±5.02 years. Sociodemographic characteristics and internet usage times are shown in Table 1.

**Table 1:** Demographic, clinical characteristics, physical examination, and treatment history of acne patients.

Variables	Category	n (%) or mean±SD
Age (years) [min–max]	–	23.37±5.02 [18–44]
Gender	Female/male	100 (81.3%)/23 (18.7%)
Body mass index (BMI, kg/m^2^) [min–max]	–	22.64±3.87 [22.6–39.8]
Education level	University and above	98 (79.7%)
	High School and below	25 (20.3%)
Place of residence	Urban/rural	118 (95.9%)/5 (4.1%)
Smoking/alcohol	Current users (yes/occasional)	21 (17.1%)/22 (17.9%)
Occupation (top 3)	Student	76 (61.8%)
	Housewife	13 (10.6%)
	Teacher	10 (8.1%)
Internet usage	4–8 h	71 (57.7%)
	8 h and above	12 (9.8%)
Acne duration	>3 years	48 (39.0%)
	1–3 years	27 (22.0%)
	<1 year	48 (39.0%)
Acne type	Inflammatory	79 (64.2%)
	Non-inflammatory	37 (30.1%)
	Nodulocystic	7 (5.7%)
Presence of scarring	Present/absent	94 (76.4%)/29 (23.6%)
Acne severity score (GAGS)	Score	14.41±7.15
Classification based on GAGS	Mild–moderate	120 (97.6%)
	Severe–very severe	3 (2.4%)
Patient-perceived severity	Mild–moderate	86 (70.0%)
	Severe (self-perception)	37 (30.0%)
Treatment history	Patients with previous treatment	83 (67.5%)
	Topical/systemic/combined	43/23/19

SD: standard deviation; GAGS: Global Acne Grading System; BMI: body mass index.

The patients’ average GAGS in the clinical evaluation was 14.41±7.15. Scars were present in 76.4% of patients (Table 1). Of the methods used by patients to treat their acne, the most frequently used method was dermatologist recommendation at 74.8%. Social media recommendations ranked second at 40.7%, followed by pharmacy recommendations at 35.0%, with AI recommendations placed fourth at 8.9%. In total, 48% of patients stated that AI use would not affect their relationship with their physician.

A total of 45.5% of participants (n=56) stated that they had used AI tools for general health purposes before consulting a physician. However, when looking specifically at dermatological concerns, 60.1% of participants had never consulted AI for acne. Notably, only a small fraction of the entire cohort (4.1%, n=5) proceeded to implement treatment recommendations received directly from AI for their acne. Notably, 67.5% of participants believe that AI recommendations should always be approved by a dermatologist. Concurrently, 39.8% (n=49) of the participants stated that they would rely on AI tools as an alternative if a dermatologist were completely unreachable. Additionally, 55.3% of participants expressed concern about the risk of AI making incorrect diagnoses, and 48.8% stated that they were reluctant to share personal data, such as photos, with these systems (Figure 1). Of those who prefer AI, 53.7% use it for “rapid and easy access,” while 52% stated that they “would never use it without consulting a doctor.”

**Figure 1:**
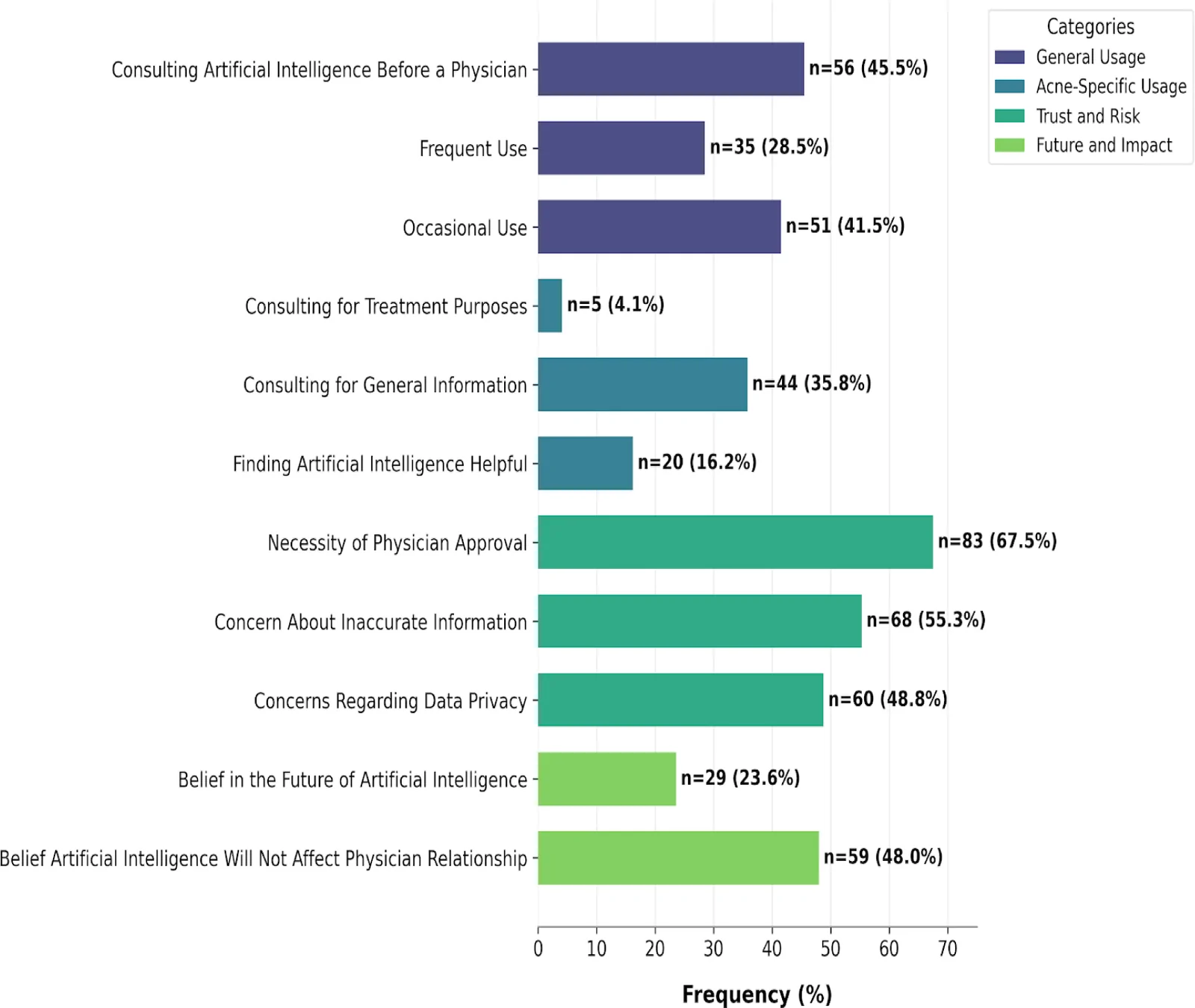
Artificial intelligence usage habits and attitudes among participants.

In the comparative analyses performed, no statistically significant difference was found between the presence of scars and AI error anxiety (p=0.29) or between gender and the level of trust in AI recommendations (p=0.56). Despite the presence of a modest positive trend between acne severity and the level of trust in AI, this relationship did not attain statistical significance (p=0.057, rho=0.172).

The relationships between participants’ use of AI for acne and demographic, clinical, and perceptual variables were evaluated using Pearson correlation analysis (Table 2). The analysis revealed a statistically significant positive relationship between AI utilization and educational status (r=0.192, p=0.034). However, no significant correlations or associations were observed between AI usage and the duration of acne (r=0.046, p=0.615) or occupational categories (p=0.274). Furthermore, when evaluating lifestyle parameters, a statistically significant positive correlation was identified between AI utilization and alcohol consumption (r=0.227, p=0.012). However, no significant relationships were observed between AI usage and smoking status (r=0.149, p=0.100) or BMI (r=-0.079, p=0.387). The analysis revealed a moderate, positive, and significant relationship between AI use for acne management and AI providing rapid and easy access (r=0.490; p<0.001).

**Table 2:** Pearson correlation analysis between artificial intelligence use for acne and clinical and perceptual variables.

Variables	Pearson’s correlation (r)	p-value
Preference for speed and convenience	0.490	<0.001*
Trust in AI recommendations	-0.455	<0.001*
Intention to implement AI recommendations	0.449	<0.001*
Belief in the future role of AI	0.392	<0.001*
Privacy concerns/data hesitancy	0.229	0.011*
Age	-0.200	0.027*
AI-related concern/anxiety	-0.108	0.237
Acne severity score (GAGS)	-0.012	0.892

*p<0.05. GAGS: Global Acne Grading System; AI: artificial intelligence.

A significant correlation was identified between trust in AI recommendations and usage (r=-0.455; p<0.001). This negative correlation indicates that as the level of trust increases based on scale scoring, the tendency to use AI also increases (1: I generally trust, 3: I don’t trust them at all). Furthermore, a weak but significant negative relationship was found between age and AI usage (r=-0.200; p=0.027), indicating that younger participants preferred AI tools more frequently. A notable finding was the absence of a statistically significant relationship between the GAGS, which reflects the participants’ clinical condition, and the use of AI for acne (r=-0.012; p=0.892).

## DISCUSSION

This study demonstrates that AI utilization among acne patients is independent of clinical severity (p>0.05), contrasting with literature that frequently links digital health usage to disease severity^7,8^. This independence may reveal a critical behavioral reality unique to acne patients, suggesting that the subjective motivation to engage with digital tools does not always correlate with objective clinical scores. Even individuals with clinically mild acne might experience an immediate desire for instant information, which could potentially prompt them to utilize AI interfaces. Regardless of clinical status, adoption is primarily driven by the patient’s established technological approach and the demand for rapid accessibility. Consequently, patients perceive AI not as a medical necessity, but as a modern technological tool integrated into their digital routines.

Despite the proliferation of digital health platforms^9^, the vast majority of acne patients in our study adhered to dermatologist recommendations. While nearly half sought AI advice prior to consultation, fewer than 5% followed it. This finding aligns with existing literature showing that 84.4% of patients prefer a dermatologist’s diagnosis over AI—even when accuracy is equal^10^ and that exclusive trust in AI applications is limited to just 1.5%^11^. Collectively, these data underscore that patient trust in physicians remains paramount.

Prior research in skin cancer screening underscores that while patients value AI for its efficiency and accuracy, they consider physician validation essential to preserving the doctor–patient relationship^12,13^. While a majority of participants highlighted the potential advantages of AI in healthcare, including enhanced diagnostic accuracy, efficiency, time-saving benefits, and personalized care, a significant proportion of patients expressed concerns regarding data security, reliability, and the potential loss of human interaction in medical settings^14^. Mirroring these insights, 53.7% of our participants prioritized AI for its rapid accessibility, despite nearly half expressing concerns over diagnostic errors and data privacy. Crucially, the persistence of AI utilization despite these apprehensions suggests that patients often perceive immediate digital benefits, such as instant guidance, as outweighing potential data security risks.

While innovative telemedicine models can mitigate limited dermatological access^15^, approximately 40% of our participants would rely on AI if a physician were unreachable. However, the “black box” nature of deep learning—the opacity of its predictive mechanisms—directly contrasts with the transparency required in standard clinical consultations^15,16^. Consequently, achieving diagnostic transparency is as vital as ensuring accuracy; AI systems must provide explainable rationales to preserve the foundational trust within the physician–patient relationship.

The findings of the present study are limited in their comparability with existing medical literature, particularly regarding acne treatment, due to a lack of data on patients’ perceptions of AI. However, a more thorough examination of the available research reveals that while patients have a rudimentary understanding of the AI concept, they possess limited knowledge of its clinical applications. Demographic trends indicate that AI literacy is higher among males, younger individuals, and those pursuing higher education in science^17^. Our finding that younger age is associated with AI use aligns with the notion that younger patients are more open to technology for addressing health concerns. However, this trend carries risks, such as the potential misdirection of clinically severe acne cases toward inadequate or incorrect AI-recommended treatments, primarily due to their ease of accessibility.

Notably, a patient’s familiarity with technology does not invariably correlate with their level of trust. Instead, patients emphasize the importance of AI operating under the direct supervision of a clinician, serving as a complementary tool rather than a substitute^17^. Indeed, the results of the present study indicate that 55% of respondents expressed concern regarding erroneous AI recommendations, while 67% considered clinician validation to be essential.

While literature suggests AI can enhance patient–clinician interactions by encouraging professional care^12,17,18^ and reducing superfluous visits^17,19^, our findings indicate that AI utilization does not actively influence the patient–physician relationship. This neutral impact aligns with previous data showing that online health information acquisition had a completely neutral effect on the patient–physician dynamic for 81.4% of individuals^20^.

Limitations of our study include the limited sample size, lack of demographic diversity, and the homogeneity of the study group, which primarily consisted of young university students. It is important to note that some patients’ views may be based on media perceptions or assumptions rather than actual experiences, as they lacked prior experience with AI for acne management. Furthermore, the specific AI tools or platforms utilized by the patients were not identified, precluding an analysis of how distinct algorithmic interfaces or functional capabilities might influence patient trust and satisfaction. Given that different AI modalities can uniquely influence patient trust and perception, explicitly categorizing these platforms remains a critical requirement for future research. Finally, as the study was conducted in a clinical setting, it did not account for individuals who exclusively self-medicate or do not seek professional dermatological consultation.

While further research is needed, the findings of this study have significant implications for medicine as a whole. As Clark and colleagues emphasized, AI should be used cautiously, and trends in this rapidly evolving field must be closely monitored^21^. Furthermore, while the potential benefits of AI are promising, it is imperative to address challenges such as data privacy, ethical concerns, and the necessity for ongoing collaboration between AI developers and medical professionals^22^.

The outcomes of this study offer three primary insights for clinical practice: first, dermatologists should be aware that their acne patients may turn to AI tools regardless of their clinical presentation; second, it reassures dermatologists that AI acts as an initial consultation mechanism rather than a physician replacement; and third, it underscores the importance for dermatologists to guide their acne patients in digital health literacy, combining the “speed” advantage offered by AI with professional “oversight.” In conclusion, the future of AI in dermatology—specifically in acne management—depends not only on technical proficiency but also on the integration of these tools into clinical workflows in a manner that is physician-controlled, transparent, explainable, and aligned with patient values.

## Data Availability

The datasets generated and/or analyzed during the current study are available from the corresponding author upon reasonable request.
